# Protein Kinases and Their Inhibitors in Pluripotent Stem Cell Fate Regulation

**DOI:** 10.1155/2019/1569740

**Published:** 2019-07-24

**Authors:** Jungwoon Lee, Young-Jun Park, Haiyoung Jung

**Affiliations:** ^1^Environmental Disease Research Center, Korea Research Institute of Bioscience and Biotechnology (KRIBB), 125 Gwahak-ro, Yuseong-gu, Daejeon 34141, Republic of Korea; ^2^Immunotherapy Research Center, Korea Research Institute of Bioscience and Biotechnology (KRIBB), 125 Gwahak-ro, Yuseong-gu, Daejeon 34141, Republic of Korea

## Abstract

Protein kinases modulate the reversible postmodifications of substrate proteins to their phosphorylated forms as an essential process in regulating intracellular signaling transduction cascades. Moreover, phosphorylation has recently been shown to tightly control the regulatory network of kinases responsible for the induction and maintenance of pluripotency, defined as the particular ability to differentiate pluripotent stem cells (PSCs) into every cell type in the adult body. In particular, emerging evidence indicates that the balance between the self-renewal and differentiation of PSCs is regulated by the small molecules that modulate kinase signaling pathways. Furthermore, new reprogramming technologies have been developed using kinase modulators, which have provided novel insight of the mechanisms underlying the kinase regulatory networks involved in the generation of induced pluripotent stem cells (iPSCs). In this review, we highlight the recent progress made in defining the roles of protein kinase signaling pathways and their small molecule modulators in regulating the pluripotent states, self-renewal, reprogramming process, and lineage differentiation of PSCs.

## 1. Introduction

Pluripotent stem cells (PSCs) have unique properties allowing them to undergo unlimited self-renewal and retain pluripotency to differentiate into any cell type in the developing organism, providing a precious source of cells for applications in regenerative medicine [1, 2]. PSCs were initially established from developing blastocyst-stage preimplantation embryos as embryonic stem cells (ESCs) [3, 4] and can now be derived from somatic cells as induced pluripotent stem cells (iPSCs) by the ectopic expression of a combination of four transcription factors: Oct4 (octamer-binding transcription factor-4), Sox2 (sex-determining region Y-box 2), Klf4 (Kruppel-like factor-4), and c-Myc (c-myelocytomatosis), collectively known as OSKM Yamanaka factors [2, 5, 6]. The technology of generating iPSCs represents a major breakthrough for the fields of stem cell biology and regenerative medicine [2, 5, 6] and is becoming more powerful along with recent advanced achievements in sophisticated technologies of CRISPR-Cas9-mediated gene-editing systems and differentiation methods such as the generation of organoids with a three-dimensional architecture [7].

PSCs are classified into molecularly distinct “naïve” and “primed” pluripotent states based on their potential to develop into the germline lineages *in vivo* and their growth features *in vitro* [8]. Primed PSCs have self-renewal ability and differentiation potential into the three germ layers *in vitro*, similar to that of naïve PSCs, but cannot generate germline-competent chimeras *in vivo* [8]. In addition, there are substantial metabolic, transcriptional, and epigenetic differences that influence the classification of pluripotent states and cell fate [9]. Naïve PSCs such as mouse embryonic stem cells (mESCs) derived from the inner cell mass (ICM) of preimplantation blastocysts represent a developmental ground state in response to the cytokine leukemia inhibitory factor (LIF) and the inhibition of kinases including glycogen synthase kinase 3 (GSK3) and mitogen-activated protein kinase (MAPK)/extracellular signal-regulated kinase (Erk) kinase (MEK) [9]. In contrast, primed PSCs such as mouse epiblast stem cells (mEpiSCs) and human embryonic stem cells (hESCs), which are derived from the mouse postimplantation stage embryos and the human ICM of blastocysts, respectively, represent a more advanced developmental state in response to fibroblast growth factor (FGF) and transforming growth factor-*β* (TGF*β*) serine/threonine receptor kinase signals [10–12].

Although PSCs can differentiate into all kinds of cell types in an adult organism, they have limitations in their ability to develop into the extraembryonic placental tissue *in vivo* [13]. Recent studies identified the extended pluripotent stem (EPS) cells or expanded potential stem cells (EPSCs) which can generate both embryonic and extraembryonic lineages *in vivo* following injection of a single cell [14–16]. LCDM condition consisting of human LIF (hLIF), GSK3 inhibitor CHIR99021, and two small molecules, (S)-(+)-dimethindene maleate and Minocycline hydrochloride, supports the generation of both mouse and human EPS cells with extended developmental potentials [14]. In contrast, the hLIF and chemical cocktail of six inhibitors (CHIR99021, MEK1 inhibitor PD0325901, JNK inhibitor VIII, p38 inhibitor SB203580, Src kinase inhibitor A-419259, and tankyrase inhibitor XAV939) facilitates the generation of mouse EPSCs which can be readily differentiated to trophoblast stem (TS) and extraembryonic endoderm stem (XEN) cells *in vitro* [15, 16].

**Figure 1:**
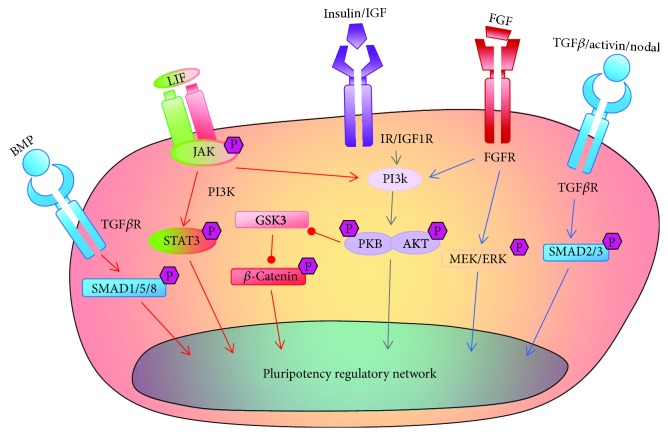
The core protein kinase signaling pathways for regulating pluripotency. LIF signaling in mESCs activates JAK/STAT3 to induce target genes essential for the naïve pluripotency regulatory network. In contrast, FGF/Erk signaling in hESCs maintains the primed pluripotent state. TGF*β*/activin/nodal signaling is essential for maintaining the primed pluripotency, whereas BMP signaling is involved in maintaining the naïve pluripotency with LIF. PI3K/AKT signaling can be activated in both mESCs and hESCs, which maintains pluripotency, indicated by a gray arrow. Red and blue arrows represent naïve and primed pluripotency activation, respectively.

The acquisition and maintenance of pluripotency are intrinsically associated with the core regulatory networks of particular transcription factors such as Oct4, Sox2, and Nanog [2] and are tightly controlled by signaling pathways that regulate reversible posttranslational modifications, especially protein phosphorylation [17]. Several protein kinases are well established to regulate the fate of PSCs, including those involved in the LIF/Janus kinase (JAK)/signal transducer and activator of transcription 3 (STAT3) axis, FGF/Erk signaling, phosphoinositide 3-kinase (PI3K)/AKT serine threonine kinase/mammalian target of rapamycin (mTOR) signaling, Wnt/GSK3 signaling, and TGF*β* serine/threonine receptor kinases signaling [18] (Figure 1).

Although the human kinome consists of 518 protein kinases that comprise 1.7% of human genes [19, 20] and protein kinases are known to regulate many cellular processes, including cell cycle progression, proliferation, metabolic homeostasis, aging, and development [21, 22], the detailed roles of the protein kinase signaling networks that modulate the self-renewal and pluripotency of PSCs remain poorly understood. In this review, we discuss the recent knowledge accumulated on the significant roles of these protein kinase signaling pathways and their small molecule modulators in regulating the pluripotent states, self-renewal, reprogramming process, and lineage differentiation of PSCs.

## 2. Kinase Signaling Pathways in Pluripotency and Self-Renewal

### 2.1. Mitogen-Activated Protein Kinase/Extracellular Signal-Regulated Kinase Pathway

The MAPK/Erk signal transduction cascade transduces the environmental signal of growth factors such as FGFs, and the optimal level of Erk signaling is critical for self-renewal and pluripotency [23]. FGFs and their tyrosine kinase receptors control diverse cellular processes, including growth, survival, migration, and differentiation [24]. The FGF signals are further relayed by four different pathways such as JAK/STAT, phosphoinositide phospholipase C (PLC*γ*), PI3K, and Erk. Autocrine FGF4-Erk1/2 signaling has an important role in promoting the transition from a naïve to a primed state and stabilizing the primed cell state [24]. Moreover, the FGF4-Erk1/2 signaling pathway regulates the pluripotent versus a differentiated state in mESCs and may be counterbalanced by the LIF and bone morphogenetic protein (BMP) signals that promote a naïve ground state [24]. LIF supports the pluripotency and self-renewal of mESCs through the receptor-mediated stimulation of JAK and activation of STAT3 [25]. LIF also activates the MEK cascade, whereas inhibition of Erk promotes the self-renewal response [21]. Indeed, LIF-JAK-STAT3 inhibitors, ruxolitinib and tofacitinib, promote a primed status, while the FGF receptor (Fgfr) inhibitors, PD173074 and AZD4547, or the MEK1/2 inhibitors, PD0325901 and PD184352, promote a naïve status [26].

However, the pharmacological inhibition of Erk signaling by PD0325901 was found to be insufficient to maintain mESCs over the long term or clonally without LIF supplementation, implying that there are alternative signaling pathways associated with pluripotency [27, 28]. Chemical inhibition of RSK1 which is the negative regulator of Erk1/2 expedites mESC lineage specification, indicating that Erk1/2 activation influences the dynamics of conversion from naïve pluripotency [29]. In addition, Erk5 signaling sustains mESCs in the naive state and suppresses progression toward a primed state and neuroectoderm differentiation [26].

Although hESCs show increased STAT3 phosphorylation in response to exogenous LIF, LIF addition does not seem to enhance self-renewal in culture [30]. It is well known that FGF2 (or basic FGF)/Erk signaling in hESCs maintains the primed pluripotent state and blocks neuronal, trophectoderm, and primitive endoderm differentiation both directly and indirectly via activin/nodal induction [24, 31]. In addition, extrinsic FGF2 signal can directly regulate Nanog expression [32] and stimulate the Ras paralog, NRas, which is linked to the MAPK pathway [33], thereby sustaining human PSC (hPSC) pluripotency. Interestingly, Erk1/2 inhibition establishes the naïve ground state of adherent hPSCs [34] but leads to a loss of the pluripotent phenotype in the suspension culture of hPSCs, suggesting that Erk is involved in a different mechanism for the suspension environment [35].

### 2.2. Glycogen Synthase Kinase 3 Signaling Pathway

Wnt signaling via *β*-catenin is a key pathway involved in embryonic development and has proven to support the pluripotency of both mouse and human ESCs [36]. Activation of Wnt signaling by inhibition of GSK3 synergizes with the activation of JAK/STAT signaling by recombinant LIF to enhance self-renewal and inhibit spontaneous differentiation of mESCs [37, 38]. Additionally, the GSK3 inhibitor CHIR99021 exhibited a short-term stimulatory effect on the self-renewal of mESCs without additional LIF and serum, and this inhibition was sufficient to maintain mESCs in combination with two other kinase inhibitors such as PD184352 for ATP noncompetitive MEK1/2 and SU5402 for Fgfr tyrosine kinase [28, 34]. GSK3 inhibition induces the stabilization of *β*-catenin, leading to long-term self-renewal primarily by abrogating function of the T-cell factor 3 (Tcf3) transcription factor which acts as a repressor on the pluripotency network, such as Oct4, Sox2, and Nanog [39, 40], and stimulates differentiation by activation of Tcf-*β*-catenin target genes, such as brachyury [41]. *β*-Catenin can also physically interact with Oct4 resulting in the upregulation of Nanog expression in a potential Tcf-independent manner [40, 42].

Dual inhibition by CHIR99021 and PD0325901, so-called two inhibitor (2i), can drive self-renewal and inhibit differentiation and conversion of naïve mESCs [34]. In contrast, Wnt inhibitors, such as IWP2 which blocks the secretion of Wnt ligands or XAV939 which promotes degradation of *β*-catenin by tankyrase inhibition, can promote the establishment of homogenous primed-state mEpiSCs [12, 43]. Similarly, Wnt signaling promotes self-renewal of naïve hESCs and Wnt inhibition induces a more primed-like intermediate state in naïve hESCs [44]. However, XAV939 facilitates stable, long-term conversion to the naïve state from primed hPSCs supplemented with hLIF-2i [45] and stabilizes the resetting process of hPSCs to a stable naïve status supplemented with t2iLGö (hLIF-2i plus protein kinase C (PKC) inhibitor Gö6983) following histone deacetylase (HDAC) inhibition [46]. It is supposed that dual inhibition of tankyrase (XAV939) and GSK3 (CHIR99021) in primed mEpiSCs and hESCs paradoxically increases Wnt signaling by increasing and stabilizing Axin2, leading to the formation of the *β*-catenin complex, and thereby increasing cytoplasmic retention of *β*-catenin and preventing the *β*-catenin-TCF interaction [47]. In addition, possible complex activities of tankyrase beyond Wnt signaling may further promote a human naïve ground state [45].

### 2.3. Phosphoinositide 3-Kinase Signaling Pathway

The PI3K/AKT/mTOR signaling cascade helps to regulate a variety of cellular processes, including cell proliferation, growth, survival, and metabolism. PI3K/AKT can be activated by insulin/insulin-like growth factor 1 (IGF1) in both mouse and human ESCs. mTOR is a conserved protein kinase that is active in two distinct complexes, mTORC1 and mTORC2. The genome-wide CRISPR-KO screen reveals that receptor tyrosine kinase- (RTK-) mediated AKT activation and the mTORC1-negative regulators such as Tsc1/2 and Gator1 play an important role in the regulation of appropriate GSK3 activity for naïve pluripotency in mESCs. Interestingly, the loss of Tsc1/2 complex causes AKT/mTORC1-dependent GSK3 inhibition, but the loss of Gator1 complex shows the opposite phenotype of GSK upregulation [48].

In both mouse and human ESCs, this PI3K/AKT/mTOR signaling pathway is essential for maintaining pluripotency [9]. Thus, PI3K inhibition by LY294002 in mouse or human ESCs leads to a decrease in the expression level of pluripotency markers concomitant with the increase of lineage-specific genes, which together strongly induce the loss of pluripotency [49, 50]. In addition, PI3K/AKT may also be activated by FGF2 in hESCs and by the LIF/JAK pathway in mESCs [9]. Interestingly, in mESCs, the LIF-PI3K pathway induces Tbx3, which is able to activate Nanog, counteracting LIF-Erk signaling, whereas the LIF/JAK/STAT3 pathway induces Klf4, which preferentially activates Sox2 [51].

### 2.4. TGF*β* Serine/Threonine Receptor Kinase Signaling Pathway

The TGF*β* superfamily of structurally related cytokines, including TGF*β*, BMPs, and activin/nodal, plays key roles in regulating the stem cell fate. TGF*β* signaling is initiated by the binding of ligand to its serine/threonine kinase receptors and phosphorylation of the cytoplasmic signaling molecules Smad2/3 for the TGF*β*/activin/nodal pathway or Smad1/5/8 for the BMP pathway. During embryonic development, epithelial cells undergo a morphogenetic event known as the epithelial-to-mesenchymal transition (EMT), in which the cell polarity and cell-cell adhesion are lost toward differentiation to mesenchymal cells. TGF*β* signaling can induce the EMT, which is essential for developmental processes, including mesoderm and neural tube formation [52, 53].

TGF*β* signaling is essential in sustaining the pluripotency of hESCs and mEpiSCs, and its modulation can lead to direct lineage-specific differentiation [53]. By contrast, BMPs rapidly induce differentiation of hESCs but sustain self-renewal of mESCs with LIF [54, 55]. Indeed, inhibition of Smad2/3 phosphorylation by the T*β*RI kinase inhibitor SB431542 leads to a reduction in Oct4 and Nanog expression [56] and differentiation of hESCs toward the neuroectoderm lineage [57], although Smad2/3 can cooccupy the genome with the Oct4, Nanog, and Sox2 in mESCs but SB431542 decreases only proliferation of mESCs without affecting their pluripotency [54, 58]. By contrast, inhibition of BMP signaling cannot exhibit such effects, suggesting that TGF*β*/activin/nodal signaling, but not BMP, is necessary for proliferation of mESCs [58].

### 2.5. Cyclin-Dependent Kinase Signaling Pathway

Functional screening and phosphoproteomic profiling suggested novel roles for kinase signaling pathways in modulating the stem cell fate [18]. Cyclin-dependent kinases (CDKs) directly modulate the phosphorylation and/or expression of pluripotency-associated factors such as Oct4, Sox2, and Nanog or the determination of the stem cell cycle phase [24, 59, 60]. The activities of CDKs can also influence the modulation of signaling pathways that regulate self-renewal and differentiation [18]. In addition, mitotic Aurora kinase modulates the phosphorylation and degradation of p53 to promote pluripotency [61] and also regulates Oct4 function in mouse PSCs (mPSCs) [62]. Therefore, the cell cycle progression of PSCs is inherently associated with the maintenance of pluripotency and lineage commitment [18], implying that cell cycle kinases might be involved in the decision of PSC fate as key pluripotency regulators.

## 3. Kinase Inhibitors and the Naïve Primed Pluripotent State Transition

Human naïve PSCs are a recently derived population from human embryos or primed hPSCs [28, 34, 36, 63–65]. Notably, hPSCs can be reset to a naïve pluripotent state following short-term expression of Klf2 and Nanog [28, 65]. LIF/2i plus Gö6983 has been used to allow for the reset cells to attain a homogeneous ground state [65]. LIF/activin/5i (2i plus three inhibitors against BRaf, SB590885; Rho-associated kinase (ROCK), Y-27632; and Src, WH-4-023) identified by high-throughput chemical screening for a kinase inhibitor library was shown to support the maintenance of a naïve pluripotent state [28]. These methods allowed for obtaining a global gene expression profile that most closely resembles the cells of human preimplantation embryos [46, 66, 67].

**Figure 2:**
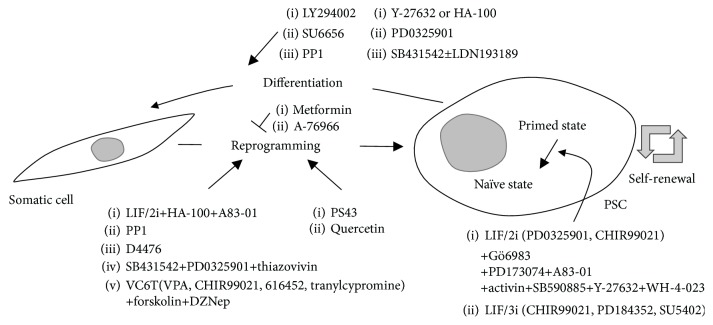
Pharmacological regulation of pluripotent stem cell fate by selective protein kinase inhibitors. Small molecule compounds modulating the activities of protein kinases facilitate the somatic reprogramming process toward the pluripotent state, promote the conversion of different pluripotent states, regulate self-renewal growth, and promote differentiation/lineage specification of PSCs.

Moreover, the LIF/2i/PD173074 tyrosine kinase inhibitor promotes the transition of human primed PSCs to naïve PSCs or their maintenance in a naïve pluripotent state, in combination with Oct4, Klf4, and Klf2 [68], and also converts mEpiSCs to a naïve pluripotent state in combination with A83-01 (inhibitor of the TGF*β*/activin/nodal pathway) [69]. LIF/3i (CHIR99021, PD184352, and SU5402) could facilitate the first isolation and establishment of germline-competent ESCs from rat blastocysts [70]. However, XMD8-85, XMD8-92, and XMD11-50, the inhibitors of Erk5 and BET bromodomain family, drive mESCs toward a primed pluripotent state [26]. These results show that small molecule compounds for kinase inhibition are capable of effectively promoting the conversion of different pluripotent states (Figure 2).

## 4. Kinase Modulators in Somatic Cellular Reprogramming

Small molecule compounds provide a useful supplement in the development of a more efficient and safer method to generate clinical-grade iPSCs [71, 72]. Selective protein kinase inhibitors facilitate the reprogramming process toward the pluripotent state by modulating the activities of protein kinases [18]. In particular, human fibroblasts can be efficiently reprogrammed to iPSCs with LIF/2i, A83-01, and HA-100 (inhibitor of protein kinases (PKs), including PKA, PKC, and PKG) [73]. The tyrosine kinase inhibitor PP1 and the inhibitor of the TGF*β*/activin/nodal pathway D4476 were shown to facilitate the reprogramming of mouse embryonic fibroblasts (MEFs) to iPSCs in the absence of Sox2 and Oct4, respectively [71, 74]. Similarly, SB431542 (an inhibitor of the TGF*β*/activin/BMP pathway including activin-like kinase (ALK) 4/5/7) is also shown to increase the efficiency of the reprogramming of human somatic cells to iPSCs in combination with PD0325901 and the ROCK inhibitor thiazovivin [75]. In addition, the generation of hiPSCs could be significantly enhanced by inhibiting the function of reprogramming barrier kinases, including p38, inositol trisphosphate 3-kinase (IP3K), and Aurora A kinase, with their chemical inhibitors [76].

The mesenchymal-to-epithelial transition (MET) is the hallmark crucial event toward the derivation of iPSCs from somatic cells, which is coordinated by repression of the EMT [77]. The TGF*β* signaling pathway plays an important role in EMT, and its inhibition can consequently enhance mouse and human reprogramming [78]. A kinome-wide RNA interference-based analysis identified protein kinases that regulate the reprogramming of somatic cells to iPSCs. Knockdown of the serine/threonine kinases testicular protein kinase 1 (TESK1) or LIM kinase 2 (LIMK2) promoted the MET transition and decreased the level of phosphorylation of the actin-binding protein COFILIN (COF) during the reprogramming of MEFs, thereby enhancing iPSC generation. Likewise, knockdown of TESK1 in human fibroblasts could also promote somatic reprogramming to iPSCs [22]. A recent study shows 315,000 single-cell RNA sequencing (scRNA-seq) profiles by a time course with two phases from either 2i or serum condition of iPSCs reprogramming from secondary MEFs. Cells show the gradual transition to either stroma-like cells or MET state, and interestingly, neural-like cells emerge from the MET region only under serum condition, suggesting that dual inhibition of MEK and GSK3 accelerates the reprogramming process and blocks the generation of neural-like off-target cells [79].

The metabolic shift from oxidative phosphorylation (OxPhos) to glycolysis is also a crucial event in somatic cellular reprogramming. Both naïve and primed PSCs show increased dependence on glycolysis under aerobic conditions with severe OxPhos suppression [2, 80]. Quercetin (an inhibitor of the mTOR, PI3K/AKT, NF-*κ*B, and tyrosine kinase pathways) stimulates glycolytic metabolism and enhances the reprogramming of human somatic cells to iPSCs [2, 81]. PS48 (a potent activator of the glycolysis-related gene pyruvate dehydrogenase kinase (PDK) 1), also facilitates human somatic cellular reprogramming [81]. However, metformin and A-769662 (the activators of adenosine monophosphate- (AMP-) activated protein kinase (AMPK) that maintains cellular energy homeostasis) provide a metabolic barrier to the reprogramming into miPSCs [82]. Collectively, these findings demonstrate that kinase signaling networks involved in the metabolic shift from OxPhos to anaerobic glycolysis are essential for the progress of induced pluripotency (Figure 2).

The chemical compounds VC6T (VPA, CHIR99021, 616452, a TGF*β* type I receptor kinase (ALK5) inhibitor and a chemical replacer of Sox2, and tranylcypromine, a monoamine oxidase inhibitor) can generate iPSCs from mouse and human somatic cells only with the exogenous Oct4 factor [81, 83]. It is first demonstrated that chemically induced iPSCs (CiPSCs) are effectively reprogrammed from mouse somatic cells at ~0.2% efficiency, by using a combination of small molecules only, VC6T supplemented to forskolin (a cAMP agonist) and 3-deazaneplanocin A (DZNep, a global histone methylation inhibitor) [71]. Furthermore, treatment of additional small molecules, including AM580 (a RAR-*α* agonist), decitabine (a DNMT1 inhibitor), EPZ004777, and SGC0946 (all DOT1L inhibitors), leads to up to a 1000-fold increase in reprogramming efficiency [84]. Interestingly, chemical reprogramming from somatic cells to CiPSCs proceeds via the formation of a XEN-like state toward pluripotency [84, 85]. Enhanced concentration of CHIR99021 up to 20 *μ*M during the early stage for 16–20 days in the chemical reprogramming process not only facilitates the generation of CiPSCs from neonatal and adult fibroblasts [71] but also is beneficial for the formation of XEN-like colonies from MEFs by initiating activation of Nanog and Sox2 in XEN-like cells, resulting in the conversion of cell fate to pluripotent cells [84]. In addition, VC6TF with 20 *μ*M CHIR99021 supplemented to AM580 and EPZ004777 promote the formation of XEN-like colonies by 2- to 3-fold [84].

A recent study has profiled the mechanistic dynamics that lead to induced pluripotency during the chemical reprogramming process using scRNA-seq. The sequential reaction of molecular dynamics toward the establishment of a full pluripotency network is analyzed, indicating that the concomitant early pluripotency and two-cell (2C) embryonic-like programs consequently accelerate the chemical reprogramming process with HDAC inhibition [85]. Until now, CiPSCs can be generated from mouse cells such as adult and neonatal fibroblasts, and adipose-derived stem cells, but not from human somatic cells [86]. Identifying alternative chemical substitutes for exogenous Oct4 factor in human cellular reprogramming requires more efforts. Generation of human CiPSCs will significantly contribute to the field of stem cell biology and regenerative medicine.

## 5. Kinase Inhibitors in Lineage Commitment and Differentiation

The balance between the self-renewal and differentiation of PSCs is crucial for the developmental process and tissue homeostasis [87]. Specific regulatory protein kinases and their inhibitors have been shown to control the intracellular signaling network for the differentiation of PSCs towards a lineage-restricted state [88]. For example, inhibition of ROCK which is involved in various physiological cellular functions including migration, apoptosis, and proliferation [89] by Y-27632 or HA-100 improves the cloning efficiencies and single-cell survival of hPSCs [40, 90] but stimulates the differentiation of mPSCs into motor and sensory neurons [91] or the cardiac lineage [92]. In addition, a high dose of nicotinamide can initiate the retinal pigment epithelium (RPE) differentiation of hPSCs by acting as an inhibitor of the ROCK and casein kinase 1 (CK1) pathways [93]. On the other hand, SB431542 promotes the differentiation of cardiomyocytes from both mouse and human PSCs by inhibition of TGF*β*/activin/nodal signaling as well as enhances the differentiation of neural progenitor cells from hPSCs in combination with the LDN193189 BMP pathway inhibitor [94, 95].

PSCs have characteristic protein-protein networks that change dynamically during differentiation [55]. A phosphoproteome analysis by stable isotope labeling by amino acids in cell culture- (SILAC-) based quantitative MS [96] reveals the phosphorylation dynamic changes during hESC differentiation by BMP induction with respect to the interplay of differentiation signaling pathways and kinase activities. Using these data to predict kinase-substrate relationships, CDK1/2 is identified to play a central role in controlling self-renewal and lineage specification, and phosphorylated forms of Sox2 are able to regulate its transcriptional activity through SUMOylation, suggesting that prevalent kinases control the activity of pluripotency-associated factors as well as cell-cycle progresses, both of which are characteristics of hESCs [55].

The Src family of nonreceptor tyrosine kinases plays an important role in diverse cellular regulation of adhesion, proliferation, growth, and survival [97, 98]. mESCs express seven Src family kinases (SFKs), but three factors including Hck, c-Src, and Fyn exhibit constitutive activity in mESC self-renewal in the presence of LIF and serum [99]. On the other hand, there are eleven SFKs in the human genome, but Lck and c-Yes may influence self-renewal of hESCs, while c-Src and Fyn may be related to differentiation [98]. Furthermore, the potent pan-SFK inhibitor A-419259 increases Oct4 and alkaline phosphatase (AP) activity and suppresses the differentiation of mESCs to embryoid bodies (EBs) while maintaining pluripotency despite the absence of LIF and retains colony morphology and pluripotency marker TRA-1-60 expression of hESCs despite culture under differentiation conditions [98, 99]. In contrast, the potent ATP-competitive SFK inhibitor SU6656 promotes epithelial differentiation of hPSCs by potently increasing the expression of cytokeratins 18 and 8 (K18/K8) while decreasing the expression of Oct4 [100], and another SFK inhibitor PP1 enhances multilineage differentiation of hPSCs by modulating the cell cycle and activity of retinoblastoma protein (Rb) and enriching the proportion of hPSCs in the early G1 phase of the cell cycle [101]. Together, these imply that SFKs and their inhibitors may control a molecular switch in modulating self-renewal and lineage specification.

## 6. Conclusions

This review summarizes the role of pluripotency-associated kinases in regulating the unique characteristics of PSCs with regard to pluripotent states, self-renewal, reprogramming process, and lineage differentiation. Achieving microenvironmental control using specific kinase inhibitors can help to regulate various kinase signaling networks, which can ultimately significantly affect the expression or function of pluripotency and/or reprogramming factors that determine the PSC fate. This integration of molecular mechanisms suggests that pluripotency is largely maintained or induced through posttranslational modifications. Although different signaling pathways, including the FGF/Erk pathway, PI3K/AKT/mTOR signaling, LIF/JAK/STAT3 axis, Wnt/GSK3 signaling, and TGF*β* family, have been well characterized, the diverse novel kinase networks that modulate self-renewal and pluripotency still need to be identified, which requires more comprehensive phosphoproteomic and functional kinome analyses. It is also necessary to investigate the elaborate molecular mechanisms of distinct pluripotent states requiring kinase functions in PSCs, which will allow for the discovery of new molecular targets linked to the PSC fate. Furthermore, the use of small molecule compounds that modulate kinase activities will also help to unravel the underlying molecular mechanism of pluripotency-associated kinases, while further providing a powerful tool for modulating the PSC fate, thereby contributing to promoting the field of regenerative medicine.
